# Effects of green synthesized zinc nanoparticles alone and along with albendazole against hydatid cyst protoscoleces

**DOI:** 10.1016/j.amsu.2022.103746

**Published:** 2022-05-11

**Authors:** Mojtaba Shakibaie, Amal Khudair Khalaf, Marziyeh Rashidipour, Hossein Mahmoudvand

**Affiliations:** a Pharmaceutical Sciences and Cosmetic Products Research Center, Kerman University of Medical Sciences, Kerman, Iran; b Department of Microbiology, College of Medicine, University of Thiqar, Iraq; c Razi Herbal Medicines Research Center, Lorestan University of Medical Sciences, Khorramabad, Iran

**Keywords:** Cystic echinococcosis, Scolicidal, In vitro, Ex vivo, Apoptosis, Caspase-3

## Abstract

**Background:**

The current investigation aims to green synthesized the zinc nanoparticles (ZnNPs) using *Lavandula angustifolia* extract by microwave technique and its protoscolicidal effects alone and combined with albendazole against hydatid cyst protoscoleces.

**Methods:**

Different concentrations of the ZnNPs (50, 100, and 200 μg/ml) alone and combined with albendazole (ALZ, 100 μg/ml) were treated with hydatid cyst protoscoleces obtained from liver of infected sheep for 5–60 min *in vitro* and *ex vivo*. Eosin exclusion examination was used to assess the viability of protoscoleces. The induction of apoptosis in hydatid cyst protoscoleces was assessed by measurement of the Caspase-3 activity of protoscoleces treated with various concentrations of ZnNPs.

**Results:**

The size of green synthesized ZnNPs was ranged from 30 to 80 nm, most of these nanoparticles were between 50 and 60 nm in size. In vitro, the highest scolicidal effect of ZnNPs was observed at the concentration of 200 μg/ml, where it killed 81.6% of protoscolices. While the combination of these nanoparticles with ALZ, especially at the concentration of 200 μg/ml, completely killed the protoscolices after 10 min’ exposure. However, compared to *in vitro* assay, the drugs tested took longer to show their protoscolicidal effect.

**Conclusion:**

Based on the obtained results, ZnNPs particularly in combination with albendazole displayed the potent protoscolicidal *in vitro* and *ex vivo* as an intraperitoneal model of administration of agents to hydatid cyst treatment; nevertheless, additional investigations are mandatory to evaluate the efficacy and safety Zn NPs as a favorable protoscolicidal agent in clinical setting.

## Highlights

• ZnNPs particularly in combination with albendazole displayed the relevant protoscolicidal *in vitro* effects.
• ZnNPs particularly in combination with albendazole displayed the potent protoscolicidal *ex vivo* effects.
• ZnNPs induced the apoptosis through the activation of caspase-3 enzyme.
• Additional investigations are mandatory to evaluate the its efficacy and safety in clinical setting.

## 1 Introduction

Cystic echinococcosis (CE) also known as hydatid cyst diseases is a potentially dangerous, occasionally fatal, infection initiated by the larval forms of the *Echinococcus granulosus* (*E. granulosus)* dog tapeworm [1]. Human mostly infected by eating of food or water containing the eggs which are excreted in the stool of an infected definitive host (etc. dogs, foxes, and wolves). After hatching the ingested eggs in small intestine, and released embryos enter the wall and move via the blood circulation into different organs, particularly the liver and lungs; where grows into a hydatid cyst [2].

The clinical symptoms of hydatid cyst infection are depending on the site and dimension of the cyst. Although, the infection can be asymptomatic in earlier phases once the cysts are small; however, with increasing cyst size and consequently mechanical influence on neighboring organs, secondary infection or anaphylactic shock due to cyst rupture, and the development of fistula in vicinal tissues severe and even fatal clinical signs may be occurred [[3], [4], [5]].

CE is generally complicated to treat; however, there are some therapeutic strategies, e.g. the use of anti-infective agents (benzimidazole derivatives), percutaneous management approachs, surgery, and even “watch and wait” with no intervention [6,7]. The most common and serious complication following hydatid cyst surgery is discharge or rupture of the cyst and the dissemination of its contents (protoscoleces) which may cause secondary infection, anaphylaxis shock, and even death [8,9]. From last decades, surgeons to prevent such complications during surgery use a number of synthetic protoscolicidal drugs including ethanol, hypertonic saline 20%, betadine (povidone-iodine), silver nitrate, etc [10]. Nevertheless, recent reports confirmed that the conventional scolicidals are connected with various complications such as biliary cholangitis, sclerosing cholangitis, cirrhosis, etc [11,12]. These reasons encourage researchers for finding a new protoscolicidal agent to treat CE.

Nanoparticles (NPs) have exceptional features due to their size (1–100 nm) and also various morphological shapes that can have important applications in nanomedicine for the control and managment of diseases [13,14]. Chemical and physical methods in nanoparticle synthesis have many limitations in use due to some difficulties such as being time consuming, high toxicity, their environmental hazards, different weaknesses once applied in microbiology [15]. Today, plant-based synthesis of nanoparticles called “green synthesis” is well-known as a reliable, valuable, and ecological friendly approaches which broadly used in nanomedicine [16].

Among the many techniques for the synthesis of nanomaterials, the microwave heating technique [17], due to having some features, e.g. identical and particular heat scattering, increasing speed of reaction and consequently, reducing the time and energy required to accomplish synthesis has received considerable attention for the synthesis of nanoparticles [18].

Zinc (Zn) as a critical element is significantly complicated in the activity and function of many enzymes and macromolecules in cell growth and synthesis of DNA, RNA, proteins [19]. Although various investigations confirmed that Zn-nanoparticles (ZnNPs) have a broad spectrum of biological and therapeutics properties in drug delivery system, microbial infection, etc [20]; however, the current investigation aims to green synthesized the zinc nanoparticles using *Lavandula angustifolia* (Lamiaceae family), a plant with an extensive variety of medicinal applications, e.g. anti-inflammatory, anticancer, anti-oxidant, antiviral, antifungal, antibacterial, and anti-parasitic effects [21], by microwave technique and it's *in vitro* and *ex vivo* effects alone and along with albendazole on protoscoleces of hydatid cyst.

## 2 Materials and methods

### 2.1 Preparation and characterization of biogenic ZnNPs

The percolation method was used to extract the *L. angustifolia* materials (aerial parts) using 80% methanol for 72 h at the 21 °C. ZnNPs was obtained according to the method described elsewhere [22]. The obtained ZnNPs was characterized using the UV–visible spectrum (spectrophotometer, JENWAY 6405), an X-ray diffractometer (Philips, PW1710), scanning electron microscope (SEM, KYKY-EM3200), Fourier transform infrared spectroscopy analysis (FTIR, Shimadzu IR-470, Japan) as previously explained [22].

Crystalline structure.

### 2.2 Collection of protoscoleces and viability

Hydatid cyst protoscoleces were obtained from livers of the naturally infected sheep slaughtered at Khorramabad abattoir, Iran. The protoscoleces were used as 5 × 10^3^ protoscoleces per ml of normal saline with at least 90% viability at eosin exclusion test [23].

### 2.3 *In vitro* protoscolicidal activity

After adding 0.5 ml of the protoscoleces solution to the laboratory tubes, 0.5 ml of ZnNPs at the concentrations of 50, 100, and 200 μg/ml alone and combined with albendazole (50 μg/ml) was put to the tubes and were keep warm for 5, 10, 20, 30, and 60 min. Once the incubation time was completed, 50 μl of 0.1% eosin stain (Sigma-Aldrich, St. Louis, MO, USA) was added to the remaining protoscoleces and smears were obtained on a glass slide, protected with a cover glass, and examined by a light microscope. The mortality rate of protoscoleces was recorded by counting 100 perotoscoleces; whereas normal saline and Ag nitrate were considered as negative and positive control [24].

### 2.4 Eosin exclusion test

**Fig. 1:**
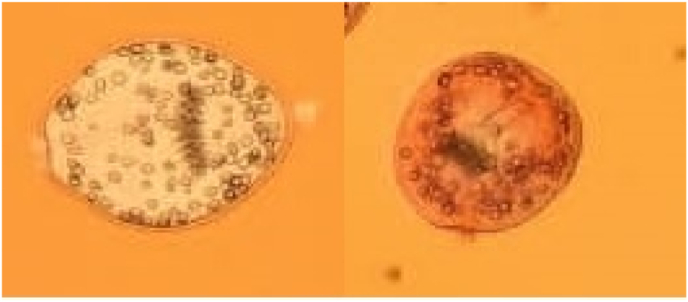
Dead (left) and live (right) protoscoleces after exposure with zinc nanoparticles.

This test was utilized to calculate the mortality rate of protoscoleces. This technique is based on the permeability of 1% eosin dye solution in the protoscoleces as well as the flame cell motility. So that the dead cells turn red; but the live protoscoleces had no color, muscular activities, and flame cell activity (Fig. 1).

### 2.5 *Ex vivo* protoscolicidal activity

To perform the *ex vivo* protoscolicidal effects of ZnNPs, almost half of the contents of liver fertile hydatid cysts was aspirated to assess the mortality rate of protoscoleces using eosin test. ZnNPs at the doses of 50, 100, and 200 μg/ml alone and along with albendazole (50 μg/ml) were inoculated into the cysts. Some of the cyst fluid comprising protoscoleces was aspirated at 5, 7, 10, 15, 20, 30, 40, and 60 min and were mixed by 0.1% eosin. Finally, the mortality rate of protoscoleces was carried out exactly like the *in vitro* assay [25].

### 2.6 Evaluating the caspase-3 like activity of ZnNPs-treated protoscoleces

To evaluate the Caspase-3 like activity of protoscoleces cured with ZnNPs we used the colorimetric protease technique (Sigma, Germany) according to the manufacturer guidelines. This assay was carried out based on the color spectrophotometric amount formed by the discharge of a molecule (pNA attached to the substrate) in the activity of the enzyme caspase-3. To do this, the protoscoleces were treated with ZnNPs for two days and were centrifuged at 800 rpm for 5 min at 4 °C, after lysing the sediment, it was centrifuged at 18,000 rpm for 12 min. At the next step, five μl of upper phase was mixed with buffer (85 μl) and caspase 3 (pNA-DEVD-Ac) solution (10 μl) and was keep warm for 120 min at 37 °C. Finally, the absorption of mixture was examined at 410 nm with the ELISA reader [26].

### 2.7 Statistical analysis

All examinations were carried out in triplicate. The results of were analyzed by SPSS. 25.0 software. Statistical examinations such as One Way ANOVA and *t*-test were also employed to estimate the differences between tested groups. P < 0.05 was reflected statistically significant.

## 3 Results

### 3.1 Characterization of ZnNPs

The characterization of the Zn NPs synthesis was shown in **Suppl 1**. UV–vis spectral analysis displayed the maximum absorption in the range of 230–330 nm. The green synthesized ZnNPs revealed a spherical shape with some masses of different dimensions. Although the size of the ZnNPs ranged from 30 to 80 nm, most of these nanoparticles were between 50 and 60 nm in size.

### *3.2* *In vitro* protoscolicidal effects of ZnNPs

**Fig. 2:**
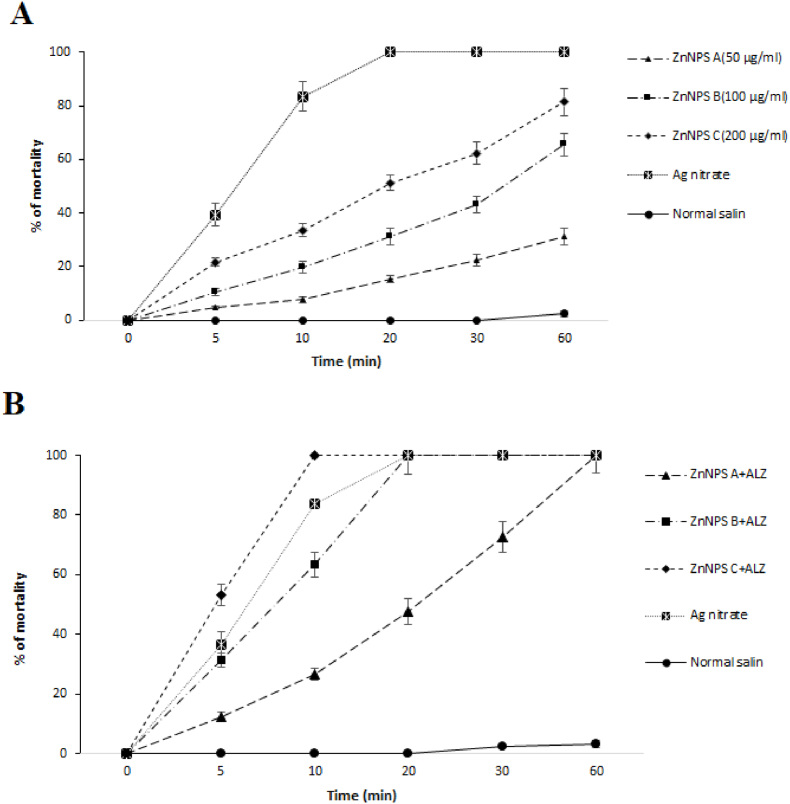
In vitro scolicidal potential of some concentrations of ZnNPs alone and along with albendazole (ALZ, 50 μg/ml) on protoscoleces of hydatid cyst protoscoleces in various incubation times compared with the control groups. Results are displayed as Mean ± SD (n = 3).

Fig. 2 shows the effects ZnNPs alone and along with ALZ on the hydatid cyst protoscoleces. Based on the obtained results ZnNPs especially when combined with ALZ represented the potent scolicidal effects compared with the control group (p < 0.001). The highest scolicidal effect of ZnNPs was observed at the concentration of 200 μg/ml, where it killed 81.6% of protoscolices. While the combination of these nanoparticles with ALZ, especially at the dose of 200 μg/ml, absolutely exterminated the protoscoleces after 10 min’ exposure.

### 3.3 *Ex vivo* effect on protoscoleces

**Fig. 3:**
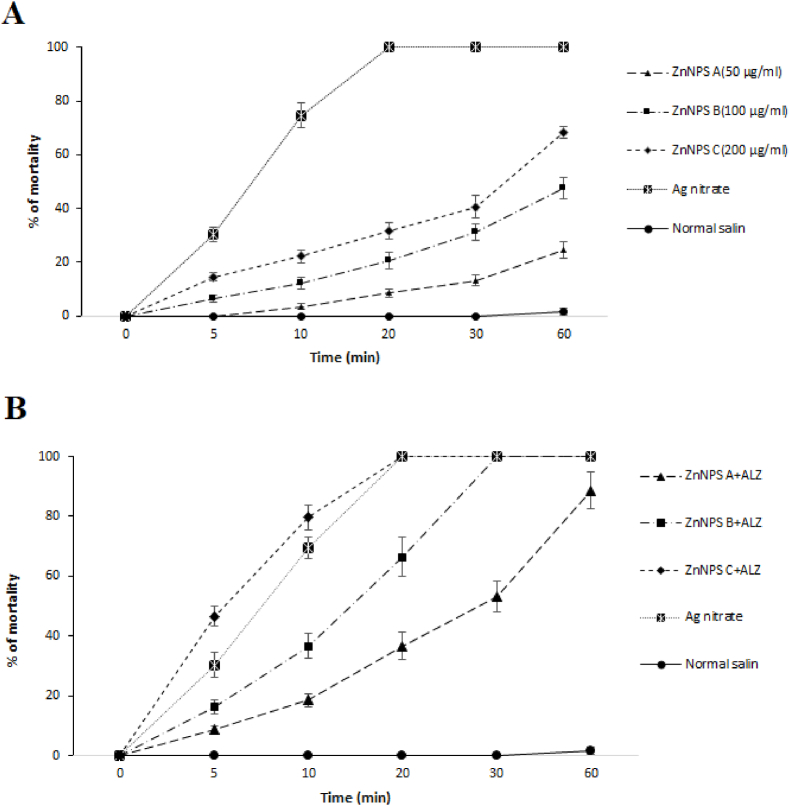
Ex vivo scolicidal potential of some concentrations of ZnNPs alone and along with albendazole (ALZ, 50 μg/ml) on protoscoleces of hydatid cyst protoscoleces in various incubation times compared with the control groups. Results are displayed as Mean ± SD (n = 3).

As shown in Fig. 3, inoculation of the ZnNPs especially when combined with ALZ into hydatid cysts considerably increased the mortality of protoscoleces. However, compared to *in vitro* assay, the drugs tested required more time to display its scolicidal effect. So that, ZnNPs at 200 μg/ml killed about 68.3% of protoscoleces after 60 min exposure; on the other hand, ZnNPs at the concentration of 200 μg/ml along with ALZ killed 100% of protoscoleces after 20 min exposure.

### 3.4 The stimulation of apoptosis

**Fig. 4:**
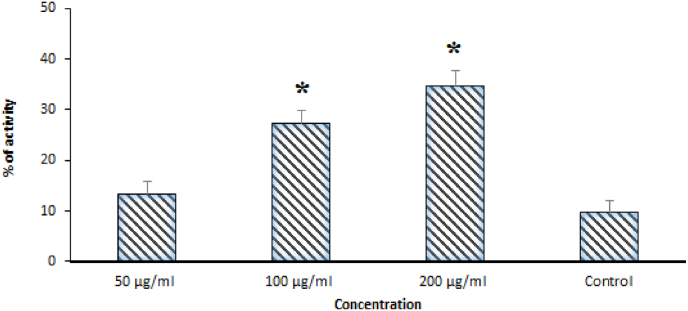
The level of Caspase-3 activity of protocoleces incubated with various different concentrations of ZnNPs compared with the control groups. Results are displayed as Mean ± SD (n = 3). *p < 0.01 displayed significant difference.

The induction of apoptosis in hydatid cyst protoscoleces was assessed by the enzymatic activity of the caspase-3. After treatment of protoscoleces with different concentrations of the ZnNPs for two consecutive days, then the changes in caspase-3 enzyme activity via calculating the concentration of released NA-p was recorded. The results showed that ZnNPs at the doses of 50, 100, and 200 μg/ml, dose-dependently stimulated the caspase-3 enzyme by 13.4%, 27.3%, and 34.8%, respectively (Fig. 4).

## 4 Discussion

CE is considered as one of the most extensively determined parasitic diseases which has monetary significance in animals and health concerns in humans because of the high risk of mortality [2,3]. Today, treating hydatid cysts seems complex and difficult, nevertheless, several therapeutic approaches such as the use the chemical agents such as benzimidazole derivatives, PAIR, surgery, and even “watch and wait” [27]. The most important complication during surgery of hydatid cyst is, rupture and leakage of protoscoleces into cyst and subsequent dissemination of them into the patient's abdominal cavity, which leads to serious complications [28]. Studies reported that the conventional scolicidals had various complications such as biliary cholangitis, sclerosing cholangitis, cirrhosis, etc [[10], [11], [12]]. These reasons encourage researchers for introducing a novel scolicidal drug to treat CE.

Based on the obtained results ZnNPs especially when combined with ALZ represented the potent scolicidal effects compared with the control group (p < 0.001). The highest scolicidal effect of ZnNPs was observed at the concentration of 200 μg/ml, where it killed 81.6% of protoscolices. While the combination of these nanoparticles with ALZ, especially at the concentration of 200 μg/ml, absolutely destroyed the protoscolices after 10 min’ exposure. Ex vivo results showed that the drugs tested required more time to display its scolicidal effect. So that, ZnNPs at 200 μg/ml killed about 68.3% of protoscoleces after 60 min exposure; on the other hand, ZnNPs at the concentration of 200 μg/ml along with ALZ exterminated 100% of protoscoleces after 20 min exposure.

With respect to the antimicrobial effects ZnNPs, investigations have exhibited the considerable effects of these ZnNPs against a wide range of bacterial (Gram-positive, Gram-negative), viral, and fungal pathogenic strains [[29], [30], [31], [32]]. Regarding the anti-parasitic effects of these nanoparticles, in the *in vivo* anticoccidial study conducted by Dkhil et al. (2015) the results showed that the treatment of infected mice with *Eimeria papillata* with ZnNPs at the dose 10 mg/kg/day ZNPs for 5 days, result in a significant reduction to 12.5 × 10^3^ oocysts, in the excretion of oocysts and inflammation in mice intestine treated with ZNPs [33]. Another study conducted by Nazir et al., 2019, *the in vitro* findings demonstrated that after exposure of promastigotes of *Leishmania tropica* with various concentration of ZnO-based nano-formulations for 24 h, the viability rate was considerably declined in Trypan blue assay with IC_50_ values varying from 0.012 to 0.084 μg/ml through the stimulating the creation of reactive oxygen species (ROS) [34]. Jan et al. also have reported the *in vitro* effects of biogenic synthesized ZnO NPs against the amastigote and promastigote forms of *L. tropica* (KWH23) with IC_50_ value of 51 μg/ml and 48 μg/ml, respectively; where, at concentration of 400 μg/ml markedly increased the mortality of promastigote and amastigote forms by 85% and 81%, respectively [35]. Esmaeilnejad et al. (2018) have exhibited the relevant *in vitro* wormicidal activity of commercial ZnO NPs with >99% killed 100% of *Haemonchus contortus* adult helminths at 16 ppm after 16 h incubation through oxidative/nitrosative destruction to critical molecules of the parasites [36].

By antimicrobial mechanisms of ZnNPs, studies have shown that these nanoparticles can possibly exert their antimicrobial features through some direct and indirect molecular mechanisms, e.g., disrupt cell growth, disruption of cell membrane permeability, stimulation of induced programmed cell death (apoptosis), provoking the production of H_2_O_2_ and subsequently inducing oxidative stress, and increased concentration of Zn in the environment and subsequently penetration of this element into the cell, which leads to disruption of some vital reactions of the cell [37,38].

In addition, the stimulation of programmed cell death which also called apoptosis is well-known as one of the key molecular mechanisms of tested agents. On the other hand, because caspases play a key role in apoptosis, we studied the caspase-3 like activity of protoscoleces exposed with ZnNPs through the colorimetric protease technique. The results showed that ZnNPs at the doses of 50, 100, and 200 μg/ml, dose-dependently stimulated the caspase-3 enzyme by 13.4%, 27.3%, and 34.8%, respectively. Similarly, previous studies have reported that ZnNPs significantly induced the apoptosis by upregulation caspase-3, caspase-8, caspase-9, Bax, LC3-II, Atg 5, p53, and Beclin 1 genes, increasing reactive oxygen species production, reducing the mitochondrial membrane potential in various cells such as human pulmonary adenocarcinoma cell line, gingival squamous cell carcinoma, mouse Leydig cell line, and human ovarian cancer cells (SKOV3) [[39], [40], [41], [42]]. Consequently, provoking the apoptosis may probably be suggested as one of the key antimicrobial mechanisms of ZnNPs.

Concerning the toxicity of ZnNPs, in a study conducted by Salari et al. (2017) have proven that two weeks' treatment of mice with biogenic ZnNPs (1, and 2 g/kg/day) no considerable alternation was observed in hematological and serum biochemical enzymes [22]. Namvar et al. (2015) have reported that ZnO-NPs represents different level of *in vitro* cytotoxicity in MTT assay with IC_50_ values of 5.6 ± 0.55, 11.75 ± 0.8, 17.45 ± 1.1, and 21.7 ± 1.3 μg/ml for cancer cells of WEHI-3B, CT-26, CRL-1451, 4T1, respectively; whereas had no considerable cytotoxicity on normal mouse fibroblast cells after 3 days’ exposure [43].

The main limitations of this study are the additional study of protoscolicidal mechanisms of green synthesized ZnNPs as well as its systematic toxicity in animal model. As future perspectives, since the *ex vivo* assay is a very good model for intraperitoneal evaluation of protoscolicidal agents, if the efficacy as well as the unwanted toxicity of these nanoparticles in animal models and then in the clinical phase in volunteers are confirmed, it can be hoped that this NPs alone or in combination with conventional drugs can be used as an effective protoscolicidal in hydatid cyst surgery.

## 5 Conclusion

Based on the obtained results, ZnNPs particularly in combination with albendazole displayed the relevant scolicidal *in vitro* and *ex vivo* (as useful model of intraperitoneal inoculation of tested drugs into hydatid cyst) activity; nevertheless, more investigations must be performed to study the efficiency and toxicity of ZnNPs as a promising scolicidal drug in human volunteers. These findings also revealed that despite the fact the likely protoscolicidal mechanisms of ZnNPs are not obviously understood, however, the stimulation of apoptosis through caspase-3 enzyme activation could be considered as one of the key mechanisms.

## Ethical approval

This experimental survey was approved by ethical committee of Lorestan University of Medical Sciences, Khorramabad, Iran (IR.LUMS.REC.1398.289).

## Sources of funding

None.

## Author contribution

Mojtaba Shakibaie: study concept or design, Amal Khudair Khalaf: data analysis or interpretation, Marziyeh, Rashidipour: data collection, Hossein Mahmoudvand: writing the paper.

## Registration of research studies

Name of the registry:

Unique Identifying number or registration ID:

Hyperlink to your specific registration (must be publicly accessible and will be checked):

## Guarantor

Hossein Mahmoudvand.

## Availability of data and materials

The datasets generated during and/or analyzed during the current study are available from the corresponding author on reasonable request.

## Provenance and peer review

Not commissioned, externally peer reviewed.

## Declaration of competing interest

The authors declare no conflict of interest.
