# Supplementary material for: Effects of green synthesized zinc nanoparticles alone and along with albendazole against hydatid cyst protoscoleces
Source: Ann Med Surg (Lond). 2022 May 11;78:103746. doi: 10.1016/j.amsu.2022.103746 (PMC9118496; doi:10.1016/j.amsu.2022.103746)
Supplement: Multimedia component 1 [file mmc1.pdf]

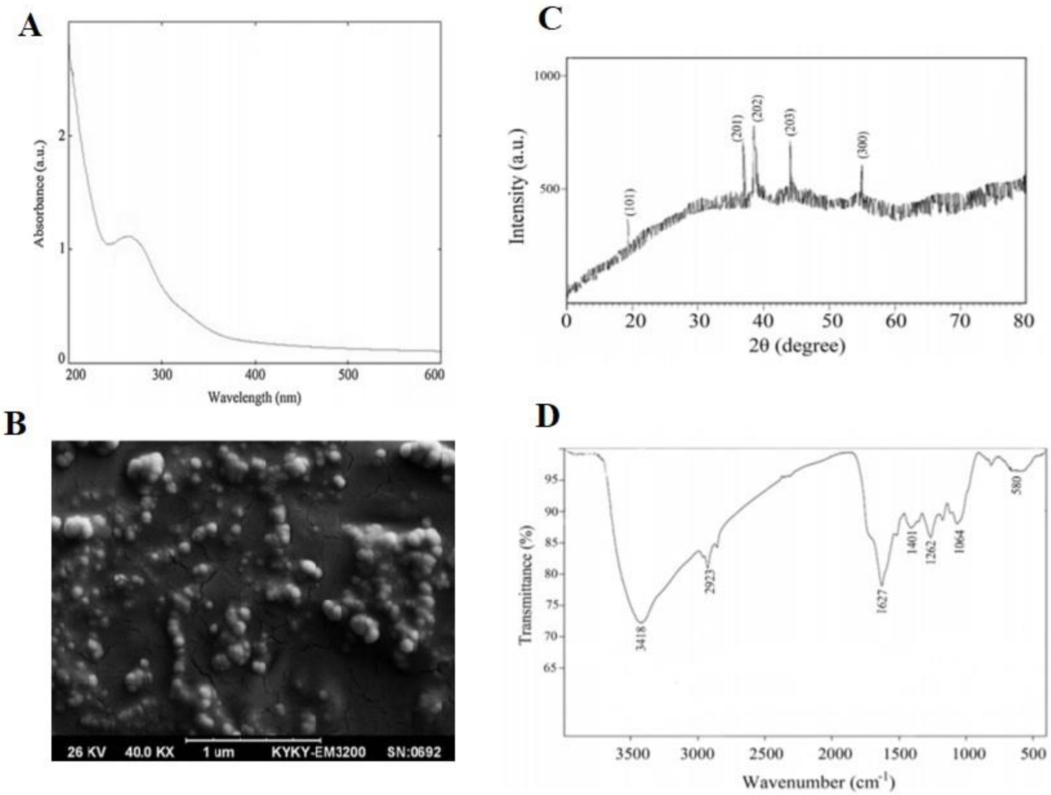


**Supplementary 1**. (A) UV–visible spectrum of the Zn NPs synthesized using *L. angustifolia* Vera. extract by microwave method. (B) SEM micrograph of the Zn NPs synthesized using *L. angustifolia* Vera. extract by microwave method. X-ray diffraction patterns of the Zn NPs synthesized using L. angustifolia Vera. extract by microwave method. (C). The FTIR spectrum of the Zn NPs synthesized using *L. angustifolia* Vera. extract by microwave method (D).
